# When Winners Become Losers: Predicted Nonlinear Responses of Arctic Birds to Increasing Woody Vegetation

**DOI:** 10.1371/journal.pone.0164755

**Published:** 2016-11-16

**Authors:** Sarah J. Thompson, Colleen M. Handel, Rachel M. Richardson, Lance B. McNew

**Affiliations:** U. S. Geological Survey, Alaska Science Center, Anchorage, Alaska, United States of America; Phillip Island Nature Parks, AUSTRALIA

## Abstract

Climate change is facilitating rapid changes in the composition and distribution of vegetation at northern latitudes, raising questions about the responses of wildlife that rely on arctic ecosystems. One widely observed change occurring in arctic tundra ecosystems is an increasing dominance of deciduous shrub vegetation. Our goals were to examine the tolerance of arctic-nesting bird species to existing gradients of vegetation along the boreal forest-tundra ecotone, to predict the abundance of species across different heights and densities of shrubs, and to identify species that will be most or least responsive to ongoing expansion of shrubs in tundra ecosystems. We conducted 1,208 point counts on 12 study blocks from 2012–2014 in northwestern Alaska, using repeated surveys to account for imperfect detection of birds. We considered the importance of shrub height, density of low and tall shrubs (i.e. shrubs >0.5 m tall), percent of ground cover attributed to shrubs (including dwarf shrubs <0.5 m tall), and percent of herbaceous plant cover in predicting bird abundance. Among 17 species considered, only gray-cheeked thrush (*Catharus minimus*) abundance was associated with the highest values of all shrub metrics in its top predictive model. All other species either declined in abundance in response to one or more shrub metrics or reached a threshold where further increases in shrubs did not contribute to greater abundance. In many instances the relationship between avian abundance and shrubs was nonlinear, with predicted abundance peaking at moderate values of the covariate, then declining at high values. In particular, a large number of species were responsive to increasing values of average shrub height with six species having highest abundance at near-zero values of shrub height and abundance of four other species decreasing once heights reached moderate values (≤ 33 cm). Our findings suggest that increases in shrub cover and density will negatively affect abundance of only a few bird species and may potentially be beneficial for many others. As shrub height increases further, however, a considerable number of tundra bird species will likely find habitat increasingly unsuitable.

## Introduction

World-wide, wildlife habitats are changing in response to climatic changes [1] and many species are responding by shifting ranges to higher latitudes or elevations as they seek suitable bioclimatic niches [2,3]. Arctic regions are projected to be disproportionately affected by climate change and many species that breed or reside in these areas are already at the limits of their geographic and climatic ranges [4,5]. As a result, many of these species are likely to witness significant changes to their habitats over time [6]. Significant climate-driven changes have already been documented in arctic regions, such as longer growing seasons, thawing permafrost, increased vegetation productivity, and changes in the frequency, intensity, and geographic scale of fires [7–10]. Numerous studies have indicated that deciduous shrubs are becoming increasingly dominant in arctic regions [11–13].

Low-growing woody vegetation is an important component of tundra flora. Dwarf shrubs (prostrate or erect shrubs <0.5 m in height) and low to tall shrubs (up to 5 m) are common in many tundra floral assemblages [14]. With climate warming and longer growing seasons, woody vegetation can expand in tundra systems in several ways. Warming experiments have shown that deciduous shrubs increase in both height and ground cover in response to increasing air temperatures [15] and taller shrub canopies may then limit the growth of other tundra plants lower in stature [11,15,16]. Shrub cover is also expected to increase as new shrubs propagate within existing shrub patches [12,15]. Finally, the shrubline may advance northward or upslope in response to climatic changes, allowing colonization by shrub-nesting birds into areas previously out of a species’ range or elevational limit [11,17]. Infilling, upslope movement, expansion along drainages, and increases in shrub height have been noted across a number of tundra ecosystems [11,13]. As shrubs become more dominant in tundra ecosystems, we expect that bird species will demonstrate variable levels of tolerance to changes. However, relatively little is known about tundra-breeding birds, making it difficult to predict how different species will respond to climate-driven changes in the structure and composition of vegetation.

Classic ecological studies from a variety of ecosystems have shown that vegetation height and density can be key predictors of avian habitat selection and species richness [18–20]. In landscapes that have been historically characterized by sparse or generally low-lying vegetation, birds that are adapted to these systems can be particularly intolerant of increasing vegetation height. For example, grassland birds are less likely to use grasslands when woody vegetation increases at either proximate or landscape scales [21,22], and many species avoid areas with even modest increases in shrub cover [23]. For Lapland longspur (*Calcarius lapponicus*) and white-crowned sparrow (*Zonotrichia leucophrys*) breeding on Alaskan North Slope tundra, shrub height was a much stronger predictor of nest-site selection than was plant species composition; longspurs rarely nested in areas with shrubs taller than 20 cm and white-crowned sparrows selected to nest at the base of shrubs between 20–100 cm tall, regardless of plant species [24].

The goal of our study was to examine tolerance of a suite of tundra-breeding bird species to increasing shrub dominance. In our subarctic study area on the Seward Peninsula, Alaska, avian breeding habitats had previously been characterized largely on the basis of the height and dominance of shrubs [25]. Thus, we predicted that even a modest expansion of shrub height or dominance could considerably alter the composition of the avian community. We used measurements of shrub height, density, and cover to assess which components of increasing shrub dominance associated with climate warming might have the greatest effect on the future distribution and abundance of tundra bird species. By better understanding avian tolerance to existing gradients of shrub dominance, we should be better able to predict which species will be most sensitive to projected climate-driven changes to arctic habitats.

## Materials and Methods

### Study Area

**Fig 1:**
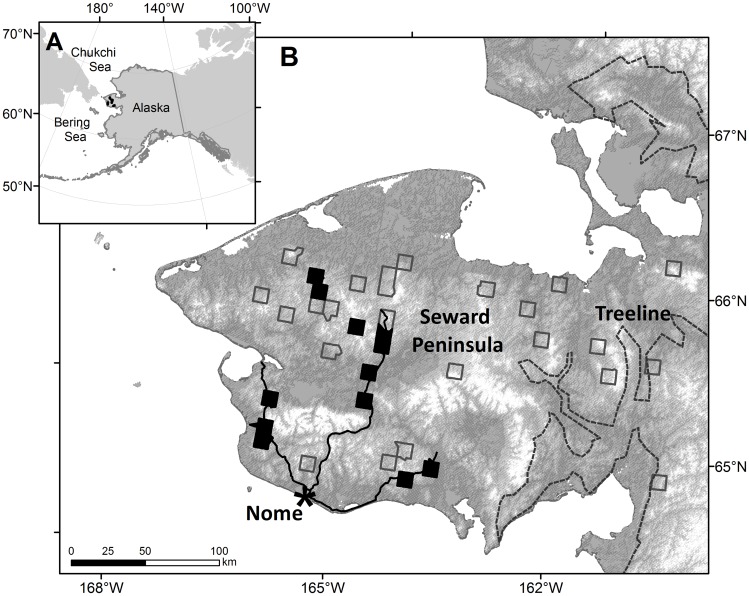
Map of Study Area and Study Sites. Study sites were located on the Seward Peninsula, in northwestern Alaska. (A) Location of the Seward Peninsula relative to Alaska and Siberia. (B) Location of all historical study blocks and the 12 (black fill) that were used for this study. Solid black lines show the road network around Nome, Alaska. Treeline (dashed line) derived from: CAVM Team 2003. Circumpolar Arctic Vegetation Map. U.S. Fish and Wildlife Service. Anchorage, AK.

We conducted our study on the Seward Peninsula, a land mass of approximately 52,000 km^2^ in northwestern Alaska that separates the Bering and Chukchi seas (Fig 1). The Peninsula is characterized by several isolated groups of rugged, glaciated mountains, oriented primarily east-west, with peaks of up to 1450 m; extensive uplands of broad, rolling hills with flat divides; interior basins; and coastal lowlands [26]. The entire Peninsula is underlain by permafrost, which thaws to varying depths during summer; lowlands have numerous thaw lakes, and drainage is primarily through many small, meandering rivers [26].

Northern and southern uplands are dominated by mesic dwarf shrub tundra, which grades into drier dwarf shrub mat tundra at higher elevations; shrub thickets of dwarf birch (*Betula nana*), willow (*Salix* spp.), and alder (*Alnus* spp.) of varying heights are common along major river drainages and along protected slopes of the foothills [25]. Poorly drained interior lowlands are dominated by wet meadows but well-drained riverbanks support shrub thickets and occasional pockets of balsam poplar (*Populus balsamifera*). The southeastern portion of the Peninsula supports fringes of boreal forest and woodlands that are common throughout interior Alaska; white spruce (*Picea glauca*) and paper birch (*Betula papyrifera*) dominate on drier sites and black spruce (*P*. *mariana*) occurs on poorly drained sites. Coastal lowlands circumscribing the Peninsula are vegetated primarily by tundra meadows [25].

The climate of the Peninsula is characterized by short, cool summers and cold, relatively dry winters, with maritime influences particularly during summer along southern coastal areas [25]. Because of its strong gradients in vegetation and climate with latitude, longitude, and elevation, the Seward Peninsula contains many diverse habitats in close proximity [25], making it an ideal laboratory for studying avian responses to habitat gradients across the boreal forest-tundra ecotone.

### Site Selection

We conducted surveys at a subset of 35 survey blocks that had been randomly selected on the Seward Peninsula and surveyed during the 1980s for bristle-thighed curlews (*Numenius tahitiensis*) and other breeding birds (C. Handel, unpubl. data; Fig 1). This study took place on a combination of public and private lands; permission was obtained from all land managers and private owners before the study commenced. Within each 100-km^2^ survey block, survey points were spaced at about 500-m intervals along ≥1 more linear or zig-zagging transects that were situated non-randomly within the block to sample available gradients of elevation and vegetation. For the current study (2012–2014), we selected 14 of the 35 study blocks that maximized accessibility and variation among sites. The number of points per study block varied from 12–58 ($\bar{x}=20.6$). We did not complete ground-based vegetation measurements for points in two survey blocks and thus dropped all bird surveys from those blocks for this analysis (Fig 1).

### Bird Surveys

Bird surveys consisted of a 10-min point count wherein observers recorded all birds detected by sight or sound within a 250-m radius [27]. We conducted surveys from 21 May to 21 June, 2012–2014. We restricted surveys to times when weather was amenable (no more than light rain and winds <20 km h^-1^). As a result of rapid weather changes, a small number of surveys (*n* = 10) were conducted in winds 20–24 km h^-1^. Most surveys were completed during morning hours ($\bar{x}=10:56$ h Alaska Daylight Time), but because of continuous daylight and the compressed breeding season at these latitudes, some surveys were conducted as late as 19:00 h when necessary and under otherwise amenable conditions (30 total surveys conducted between 16:00–19:00 h). All observers were experienced with point-count techniques and underwent 1–2 weeks of intensive training, including visual and aural identification of birds and distance estimation, before conducting surveys. We repeated surveys at some locations up to three times per season and we recorded distance to detected birds (in distance bands 0–50, 50–100, and 100–250 m) to assess detection probability [28,29]. This study did not involve endangered or protected species, nor did it involve handling or disturbance of wildlife. All protocols were approved by USGS Alaska Center IACUC #2012–9.

### Vegetation Measurements

We measured various aspects of vegetation at each survey point once during the 3-year study. From the survey point, we measured distance (m) to the nearest dwarf shrub (<0.5 m tall), low shrub (0.5–1.0 m), and tall shrub (>1.0 m) in four quadrants, based on cardinal directions (i.e. NE, SE, SW, NW). These four measurements were combined into an average distance for each shrub-height category. When there was no shrub of a specific height class within visible range of the point in any direction, we imputed a maximum value of 5 m, 110 m, and 250 m for dwarf, low, or tall shrubs, respectively, and we truncated all greater values to these distances. Maximum distances were based on natural breakpoints in data; i.e. beyond these values there were few data points.

We measured additional vegetative characteristics at 10–15 subsampling points within each bird survey area. Subsample points were placed at 5-m intervals along two or three 20-m transects with randomly selected orientations (0–359 degrees). The first transect originated at the bird survey point and the second (and third, in a few areas) was randomly placed within 250 m of the survey point. At each subsample point, we measured visual obstruction, an index of vegetation height and density, from a distance of 2 m and at a height of 0.5 m as described in Robel et al. [30]. Additionally, within a 0.5 x 0.5-m^2^ quadrat frame centered on each subsample point, we visually estimated percent cover and measured height of the tallest specimen (to the nearest cm) in each of the following categories: alder, dwarf birch, ericaceous shrub (e.g. *Empetrum* spp., *Vaccinium* spp.), herbaceous plants, lichen (e.g. *Cladonia* spp.), and willow, as well as percent bare ground. Percent cover was categorized as 0%, 1–5%, 6–25%, 26–50%, 51–75%, 76–95%, or 96–100% of the quadrat frame; we converted values to the midpoint of each category (0, 2.5, 15, 37.5, 62.5, 85, and 97.5%). At one study block with low diversity in structure and composition of plants, subsample vegetation surveys were not completed at 10 of 39 survey points. For these points, we imputed the mean value from the other 29 points in the block.

### Statistical Analysis

#### Data preparation

Several bird species were observed in high numbers during a small number of point-count surveys. In these instances, we truncated counts to the next lowest number to reduce the influence of uncommonly observed large flocks [31]: whimbrel >2 (*Numenius phaeopus*; *n* = 2), western sandpiper >4 (*Calidris mauri*; *n* = 2), bristle-thighed curlew >3 (*n* = 2), and American golden-plover >2 (*Pluvialis dominica*; *n* = 2). We had a large number of potential habitat variables and endeavored to reduce this set before commencing with analysis [32]. To reduce the number of potential covariates, we combined related measurements into single variables and eliminated variables that had limited modeling utility (e.g., where distributions were highly skewed or a majority of data points were zeros; S1 Table). We then looked for variable pairs with high collinearity (R^2^ ≥ 0.60, [31]), and removed the less informative variable or the one with more instances of collinearity (S1 Table). Ultimately, we included four covariates for bird abundance as follows. (1) Density of low to tall shrubs (excluding shrubs <0.5 m tall) was calculated using the point-centered quarter method [33]: shrubs per 100 ${\text{m}}^{2}=100/{\bar{x}}_{\text{d}}{}^{2}$, where ${\bar{x}}_{\text{d}}$ is the average distance to the nearest shrub in each quadrant. Because this calculation will generate asymptotically high values when distances to shrubs are low (e.g. 0–1 m), we added 1 m to all average shrub distances before converting. (2) Shrub height (cm) was calculated as the mean of all available height values. When a shrub from a particular group (ericaceous, willow, alder, or dwarf birch) was not present in a sample frame, height was reported as a missing value and omitted from the mean calculation; this naturally weighted the data by the more common classes of shrubs. We truncated the mean value at 100 cm, reducing 8 high values. (3) Percent shrub cover was generated by taking the sum of all types of shrub cover in each 0.5 × 0.5 m^2^ quadrat frame, then taking the average of the these sums to get an index of overall shrub cover. (4) Percent herbaceous vegetation cover was calculated as the average percent herbaceous plant cover across subsamples for each point. The maximum correlation coefficient among these four covariates was 0.53 for shrub height and shrub density, with all others considerably lower (S1 Fig). All covariates were scaled and centered for modeling (mean = 0 and SD = 1) to aid with model convergence, but are reported in original scales for figures [34].

We considered five covariates for inclusion in the detection model: wind speed ($\bar{x}=4.3$, range: 0–20 km h^-1^), hour of day (0–23), date within season (1 = May 22; $\bar{x}=20.5$, range: 1–32), observer, and average shrub height. We condensed nine observers into two groups, highly experienced (*n* = 5) and less experienced (*n* = 4), based on familiarity with birds of the area and previous experience with point counts. We conducted a series of preliminary assessments using both repeat surveys and distance-sampling methods for assessing factors that influenced detection [28,35]. Distance-sampling methods assess the likelihood of detecting a bird that is available to be detected (i.e. giving visible or aural cues), compared to repeat surveys, which do not isolate detectability from availability [28]. Thus, distance methods are preferred for assessing the effects of variables such as wind, observer skill, and vegetation density that are less closely associated with availability for detection (compared to variables such as date or time of day), and more likely to relate to an observer’s ability to detect a bird that is giving cues. Distance methods suggested limited or inconsistent support for the influence of observer skill level and shrub height, and we subsequently chose to omit these variables from consideration. We ultimately included three variables that consistently influenced detection for all species: wind speed and date (linear effects only), and hour of day (linear and quadratic effects). While biologically appropriate to consider, the quadratic term for date was problematic for many species (inducing non-convergence) and was therefore excluded. Given the short seasonal period of the surveys designed to coincide with maximum cue production, we judged the linear effect of date sufficient for consideration.

#### Model selection and fit

We modeled relationships between bird abundance and habitat characteristics with N-mixture models, which use repeated counts to assess imperfect detection while concurrently analyzing factors that influence abundance [29]. N-mixture models assume a closed population during the period between repeated visits and we met this assumption by arranging for a short duration between repeat surveys, usually 1–2 days (range 1–13 days, $\bar{x}=2.1$ days) [36,37]. We conducted bird surveys at some locations in >1 year (range 1–3, $\bar{x}=2.18$). However, we did not employ open-population models that explicitly account for interannual dynamics at a location because these parameters were not the focus of this analysis and because these models typically require large amounts of data [38,39]. Instead, for points surveyed in >1 year, we selected only the year with the largest overall count for that species (sum of all visits within a year) and omitted other years. Any additional non-independence of surveys (i.e. potential correlation of point counts within a study block) should be revealed by lack of model fit, which we formally assessed using a parametric bootstrap test [34]. Previous analysis of portions of these data also showed lack of spatial autocorrelation [40]. Finally, some species arrived on breeding grounds after surveys had begun; to avoid improper inference about habitat relationships or detection, we noted the first date that a species was observed each year and omitted any surveys conducted before that date for each year [36]. We conducted all analyses using R statistical software version 3.2.3 [41] and the package ‘unmarked’ [42].

We proceeded with model fitting in several stages. First, we used a generally parameterized model, with three detection covariates as previously described and all four habitat covariates (all included as linear and quadratic terms) for abundance, to select the better-fitting error distribution for each species, either negative binomial or Poisson, as evidenced by lower AIC values [34]. Next, we selected the formulation of each predictor that generated the best fit, as determined by lowest AIC: we considered a linear, quadratic, exponential (positive or negative), or a null relationship between abundance and each predictor variable. All other habitat variables were included as quadratic terms while a single habitat covariate was altered. The best-fitting formulations for each habitat variable were then combined to generate a single top model for each species. Because of this stage-structured process, variables may gain or lose support in the final formulation of the model; we considered a covariate to be significantly supported when the p-value estimate was <0.15, otherwise we considered it to be uninformative [43]. Finally, we assessed model fit using a parametric bootstrap method, wherein the top model was used to generate 200 simulated datasets, allowing comparison of observed and expected values via a chi-square test. In this test, a model is presumed to fit data well if the test statistic is >0.05 [34]. In some instances, the negative binomial distribution will generate very high prediction values, despite indications of acceptable model fit; in such cases, we instead generated predictions with a Poisson distribution, which is considered the better alternative [34].

## Results

During 2012–2014, we conducted surveys at 247 unique survey points within 12 100-km^2^ blocks. We observed 68 species and selected 17 of the most commonly observed species for analysis, including 12 passerines, 4 shorebirds, and 1 ptarmigan species (S2 Table). We omitted several common species from analysis because they tended to be poorly sampled by point-count methods. For example, redpolls (*Acanthis* spp.) moved erratically through survey areas in flocks that were difficult to count and were often only loosely affiliated with the habitats over which they were flying. Including within-season replicates and sites repeated in >1 year, we completed a total of 1,208 bird surveys. Once we omitted point counts that occurred before species-specific arrival dates and selected only the year with the highest total count of a given species, individual species’ datasets included 232–247 unique survey points and 487–595 point counts (Table 1). When selecting among the five potential models for each habitat covariate, in many cases there were a number of competitive models (S3 Table). In these cases, we proceeded with the selection that generated the lowest AIC. Overall, models fit data well, with all 17 species having satisfactory model fit (Table 1). For all four shorebird species, negative binomial models passed the fit test, but provided unreasonably high prediction values; thus, predictive plots were generated with a Poisson distribution.

**Table 1:** Sample dize and model fit information.

Species	Scientific Name	*n*^a^	Survey Points^b^	Point Counts^c^	Dist^d^	Shrub Height^e^	Shrub Density^e^	Shrub Cover^e^	Herbaceous Cover^e^	Model Fit Statistic^f^
American golden-plover	*Pluvialis dominica*	142	235	530	NB	Nexp	Null	Nexp	Null	0.81
Arctic warbler	*Phylloscopus borealis*	166	232	487	P	Quad	Nexp	Nexp	Quad	0.53
American tree sparrow	*Spizelloides arborea*	247	247	522	P	Quad	Quad	Exp	Null	0.82
Bluethroat	*Luscinia svecica*	207	247	585	P	Linear	Nexp	Linear	Null	0.81
Bristle-thighed curlew	*Numenius tahitiensis*	79	235	487	NB	Exp	Null	Linear	Linear	0.84
Fox sparrow	*Passerella iliaca*	600	247	580	P	Linear	Quad	Linear	Exp	0.44
Golden-crowned sparrow	*Zonotrichia atricapilla*	329	247	586	NB	Linear	Nexp	Linear	Null	0.77
Gray-cheeked thrush	*Catharus minimus*	314	247	555	P	Linear	Null	Linear	Linear	0.29
Lapland longspur	*Calcarius lapponicus*	1009	235	553	P	Quad	Quad	Null	Linear	0.45
Northern waterthrush	*Parkesia noveboracensis*	87	246	565	NB	Nexp	Null	Null	Linear	0.85
Savannah sparrow	*Passerculus sandwichensis*	741	247	595	P	Quad	Null	Linear	Nexp	1.00
White-crowned sparrow	*Zonotrichia leucophrys*	142	247	562	P	Nexp	Null	Linear	Null	0.58
Western sandpiper	*Calidris mauri*	135	235	519	NB	Linear	Null	Quad	Nexp	0.31
Whimbrel	*Numenius phaeopus*	94	247	519	NB	Quad	Null	Quad	Null	0.15
Willow ptarmigan	*Lagopus lagopus*	150	235	534	NB	Exp	Null	Null	Nexp	0.68
Wilson’s warbler	*Cardellina pusilla*	125	247	507	P	Nexp	Exp	Linear	Exp	0.07
Yellow warbler	*Setophaga petechia*	174	247	525	NB	Linear	Nexp	Linear	Exp	0.84

^a^ total number of birds included in analysis;

^b^ number of unique locations;

^c^ number of point counts (including repeat visits) used for each species, after removing early surveys and selecting one year from each survey point;

^d^ better-fitting error distribution, either negative binomial (NB) or Poisson (P);

^e^ best-approximating fit for each habitat covariate: no relationship (null), linear, quadratic (quad), exponential (exp), or negative exponential (nexp);

^f^ results of the chi-square statistic from the model fit assessment.

### Shrub Height

Average shrub height at individual survey points ranged from 0.0–94.1 cm. Survey points with high values for average shrub height (>75^th^ percentile or 14.8 cm; *n* = 62 points) occurred on 6 of the 12 sampled blocks. These points were associated with more alder cover (${\bar{x}}_{>14.8}=7.7\%$ vs. ${\bar{x}}_{\leq 14.8}=0.5\%;$ 2-sample t-test, p = 0.004) and willow cover (18.2 vs. 8.4%; p <0.001) and particularly tall alders (171 vs. 14 cm; p <0.001) and willows (40 vs. 10 cm; p <0.001) when compared with the rest of the points (*n* = 185). Survey points with the tallest shrubs were also characterized by less lichen cover (4.0 vs. 19.8%; p <0.001), increased density of low–tall shrubs (187 vs. 66 shrubs per 100 m^2^; p <0.001), and taller herbaceous plants (36 vs. 23 cm; p <0.001), but similar cover of bare ground, willow, dwarf birch, ericaceous shrubs, and herbaceous plants.

**Fig 2:**
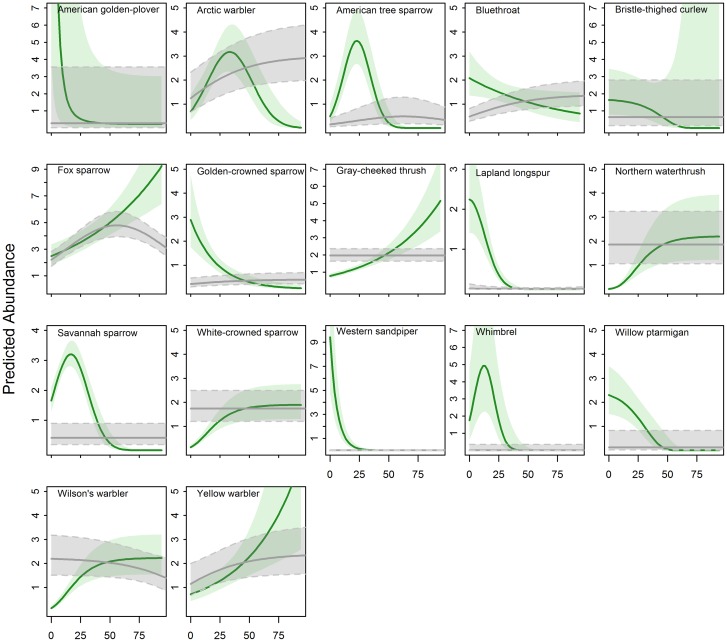
Avian Responses to Increasing Shrub Height and Density. Predicted response (abundance) of 17 species to increasing shrub height (cm; green) and density (shrubs per 100 m^2^; gray), both of which varied from 0 to 100. Predictions are based on the final model for each species with all other habitat covariates held at mean values. Shaded areas represent 85% confidence intervals.

More bird species responded to shrub height than to any other habitat variable; for 16 of 17 species, models reported a significant relationship between bird abundance and shrub height (Table 1, Fig 2; all coefficient and standard error estimates are reported in S4 and S5 Tables). Predictive models indicated that abundance of fox sparrow, gray-cheeked thrush, and yellow warbler would increase linearly with shrub height while abundance of northern waterthrush, white-crowned sparrow, and Wilson’s warbler would increase and eventually plateau with increasing shrub height (Fig 2; all scientific names reported in Table 1). Conversely, abundances of American golden-plover, bluethroat, golden-crowned sparrow, Lapland longspur, western sandpiper, and willow ptarmigan were predicted to decline with increasing shrub height, with several reaching near-zero estimates at relatively low values of shrub height (e.g. western sandpiper at ~25 cm; Fig 2). Abundances of arctic warbler, American tree sparrow, Savannah sparrow, and whimbrel were predicted to peak at low to moderate average shrub heights (33, 22, 18, and 12 cm, respectively; Fig 2). Despite model selection supporting inclusion of an exponential fit for shrub height with bristle-thighed curlew, the covariate had little support in the final model (p = 0.35, Table 1, Fig 2, S4 Table).

### Density of Low–Tall Shrubs

Low–tall shrub density (i.e. excluding dwarf shrubs) ranged from 0 to 100 shrubs per 100 m^2^ (S1 Table). Points with very high shrub densities (>75^th^ percentile or >10.3 shrubs per 100 m^2^) were observed in 10 different blocks. Points with high shrub densities had less lichen cover (${\bar{x}}_{>10.3}=3.8\%$ vs. ${\bar{x}}_{\leq 10.3}=19.8\%$, p <0.001), taller shrubs (34.5 vs. 10.9 cm, p <0.001), less ericaceous shrub cover (20.6 vs. 29.4%, p <0.001), and more alder and willow cover (7.7 vs. 0.1%, p = 0.004; and 19.2 vs. 8.0%, p <0.001) than did points with low shrub densities. Points with low and high densities of low–tall shrubs had similar values for bare ground, herbaceous plant, and dwarf birch cover.

Models indicated that abundances of seven bird species were related to shrub density; although supported in early steps of model selection, shrub density (fit with an exponential curve) was not strongly supported in the final model for Wilson’s warbler (p = 0.17). Abundances of arctic warbler, bluethroat, golden-crowned sparrow, and yellow warbler were predicted to increase with increasing shrub density; however, abundances for all four species plateaued as shrub density increased beyond ~60 shrubs per 100 m^2^ (Fig 2). Abundances of American tree sparrow and fox sparrow were best described by a quadratic response with maximum values predicted at mean values of low–tall shrub density (peaking at 65 and 52 shrubs per 100 m^2^, respectively). The top model for Lapland longspur also included a negative quadratic fit for shrub density, predicting highest abundance at 0 shrubs per 100 m^2^ (Fig 2, Table 1, S3 Table).

### Shrub Cover

Mean shrub cover ranged from 0 to 97.5% (S1 Table). Points with high shrub cover (>75^th^ percentile or >57.8% shrub cover) were observed at 7 blocks. High shrub cover was associated with taller average shrub height (21.1 vs. 15.4 cm, p = 0.01), and less bare ground (3.0 vs. 8.2%, p <0.001), herbaceous cover (27.7 vs. 39.3%, p <0.001), and alder cover (0.01 vs. 2.8%, p = 0.005). Sites with high shrub cover were also associated with considerably more ericaceous and dwarf birch cover (42.1 vs. 22.0% and 12.2 vs. 5.0%, respectively; both p <0.001).

**Fig 3:**
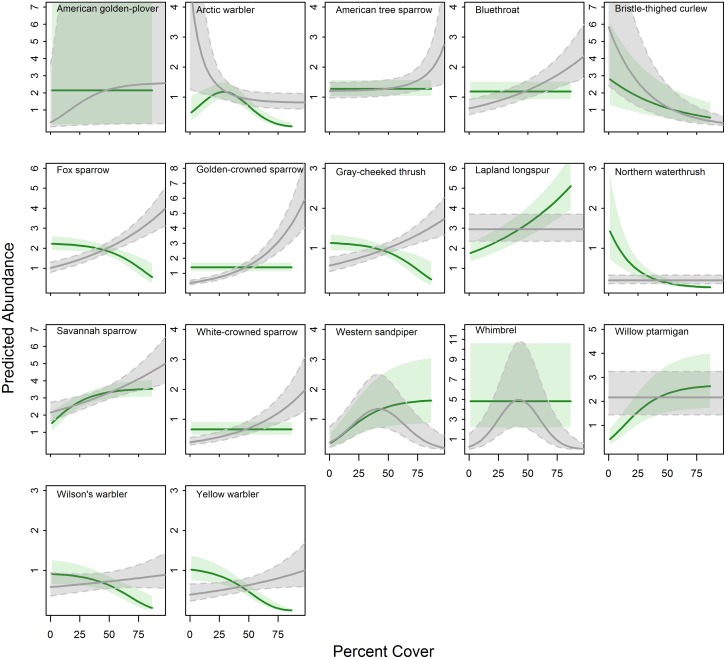
Avian Responses to Increasing Herbaceous or Shrub Cover. Predicted abundance of 17 bird species with increasing percent herbaceous cover (green) and percent shrub cover (gray). Predictions are based on the final model for each species with all other habitat covariates held at mean values. Shaded polygons show 85% confidence intervals. The x-axis represents 0–100% cover; the maximum value observed in this study during 2012–2014 for herbaceous cover was 86.5% and for shrub cover was 97.5%.

Models indicated that abundances of 13 species were associated with percent shrub cover (S4 Table). In most cases shrub cover was positively associated with abundance and maximum abundance values were predicted at or near 100% shrub cover (American golden-plover, American tree sparrow, bluethroat, fox sparrow, golden-crowned sparrow, gray-cheeked thrush, Savannah sparrow, white-crowned sparrow, and yellow warbler; Fig 3, Table 1, S4 Table). Abundances of arctic warbler and bristle-thighed curlew were predicted to decline with increasing shrub cover (Fig 3). Western sandpiper and whimbrel demonstrated quadratic responses, with maximum predictions at mean values of shrub cover (occurring at 41% and 42% shrub cover, respectively; Fig 3). The positive association between Wilson’s warbler abundance and shrub cover was not supported in the final model (p = 0.43, Fig 3, S4 Table).

### Herbaceous Cover

Average percent herbaceous cover ranged from 1.0–86.5 (S1 Table) and high values for average herbaceous cover (>75^th^ percentile or 50.6%) were observed at points within 9 study blocks. Points with high herbaceous cover had less bare ground (0.7 vs. 9.1%, p <0.001), less lichen (7.0 vs. 18.8%, p<0.001), and less ericaceous shrub cover (18.7 vs. 30.1%, p <0.001). Points with more herbaceous cover had taller herbaceous plants (32.7 vs 24.4 cm, p <0.001).

Abundance of 11 species was associated with herbaceous cover. Abundances of bristle-thighed curlew, fox sparrow, gray-cheeked thrush, northern waterthrush, and Wilson’s warbler were predicted to decline with increasing herbaceous cover. Lapland longspur, Savannah sparrow, western sandpiper, and willow ptarmigan were positively associated with percent herbaceous cover. Arctic warbler abundance was best described by a quadratic relationship with herbaceous cover, with maximum predicted abundance occurring at 29% cover. Abundances of American golden-plover, American tree sparrow, bluethroat, golden-crowned sparrow, white-crowned sparrow, and whimbrel had no significant associations with herbaceous cover (Fig 3, S4 Table).

### Detection

Predicted detection probability during average survey conditions (wind = 8 km h^-1^, date = day 20 or 11 June, and time = 10:00 h ADT) ranged from 0.09 (SE: 0.03) for whimbrel to 0.65 (SE: 0.03) for fox sparrow (average predictions for all species reported in S5 Table). Wind had a significant negative effect on detection for 9 of 17 species and a positive effect on detection for whimbrel (all coefficient estimates, standard errors, and p-values are reported in S5 Table). Date was a significant predictor for 10 species, with a negative relationship estimated for bluethroat, northern waterthrush, white-crowned sparrow, whimbrel, and willow ptarmigan and a positive relationship for arctic warbler, American tree sparrow, gray-cheeked thrush, Savannah sparrow, western sandpiper, and yellow warbler (S5 Table). Detection probability was related to time of day for five species, with models supporting a quadratic relationship for all of these species. Detection probabilities of American tree sparrow, bluethroat, fox sparrow, golden-crowned sparrow, gray-cheeked thrush, and Savannah sparrow were predicted to peak at 09:24, 09:30, 09:00, 09:06, 09:48 and 08:30, respectively.

## Discussion

The relationship between bird species richness and vertical structural complexity of vegetation in an area has long been recognized [19,44]. The nature of this relationship and ecological processes driving it are of increasing interest to ecologists attempting to predict effects of climate change on avian diversity across broad landscapes and environmental gradients [43–47]. In tundra ecosystems, shrubs of varying stature are a key component of the flora [14,48] and numerous breeding birds rely on them for forage, shelter, and nesting sites [25]. Increasing growth and dominance of shrubby vegetation are associated with warmer, longer growing seasons and possibly overall increases in productivity [10,11]. Thus, one might predict that more productive tundra systems in the future would be characterized by taller and more abundant shrubs, supporting more individual birds and a greater diversity of bird species, with perhaps the loss of a few shrub-intolerant species. Our findings, however, suggest that even species that prefer or rely on shrub habitats have limits. Therefore, identifying climate-change “winners versus losers” likely oversimplifies potential nonlinear responses. Furthermore, changes in biodiversity in tundra ecosystems over time as structural vegetation complexity increases are likely to reflect a constantly changing array of avian species.

Among 17 species considered in our study, 12 were responsive to at least one characteristic of shrubs, with predicted abundance declining either continuously or as shrubs crossed certain thresholds in height, density, or cover. Lapland longspurs prefer to nest in low-stature vegetation throughout their range [49], and the expansion of taller shrubs into herbaceous and dwarf shrub habitats is likely to have strong negative effects on their abundance in affected regions. The remaining species, however, appear to be responsive to thresholds of vegetative characteristics. For example, models predicted a rapid increase in arctic warbler abundance with increasing average shrub height, but when the height metric crossed ~30 cm, arctic warbler abundance declined. Even species that had positive relationships with shrubs often displayed plateaus where increasing shrub values eventually led to no further predicted increases in abundance (e.g., abundance of both Wilson’s warbler and northern waterthrush plateaued at ~60 cm average shrub height). As the climate continues to warm and shrub encroachment progresses, our results suggest that we will see short-term increases in abundance of many bird species, but as shrubs become increasingly dominant, many shrub-tolerant species may eventually be displaced.

Our models also suggest that bird species are responsive to different aspects of increasing shrub dominance in tundra ecosystems. For example, the top model for golden-crowned sparrow predicted a positive association with percent shrub cover and a negative association with shrub height. It may seem counterintuitive that a bird would be averse to tall shrubs, yet tolerate areas with high shrub density. In many tundra areas, however, dwarf or prostrate shrubs rarely grow taller than 0.1 m and high values of one shrub metric (e.g. cover) do not necessarily indicate association with others [14]. Given that birds responded variably to metrics of shrub cover, height, and density, it may be more difficult to predict which species will be most affected by climate-induced changes to vegetation. Will shrubs grow larger and taller or will low-growing shrub ground cover become increasingly dominant? Each of our shrub metrics can be related to one or more mechanisms of shrub expansion: increased recruitment within existing shrub patches, increased growth potential (i.e. taller or larger shrubs), or range expansion [11]. Given that studies from across northern latitudes have described increases in shrub height and cover, range expansions, infilling, and movement upslope and along water drainages [11], we believe that all of our shrub metrics (height, cover, and density of low–tall shrubs) will continue to increase as they have over the past century, thereby prompting continued and varied responses from more bird species.

Of our three shrub metrics, the average height of shrubs was negatively associated with abundance of more species than shrub density or cover. In Denali National Park in interior Alaska, Mizel et al. [17] examined how bird occupancy shifted with elevation over a 20-year time span. Among their study species, they found that arctic warbler, Savannah sparrow and golden-crowned sparrow demonstrated more dramatic shifts in occupancy and elevation than other species. Our results similarly show that these species were all responsive to increasing shrub height, again suggesting that shrub height is a particularly important driver of avian habitat selection.

A number of ecological mechanisms may cause birds to be responsive to areas with increasingly tall shrubs. Tall shrubs can outcompete other vegetation lower in stature, including low-growing shrubs, lichen, moss, and herbaceous plants [12]. This reduction in some types of low-growing ground cover around tall shrubs may deter birds that tolerate shrubs, but nest on the ground, and prefer certain types of low-growing vegetation for their nest sites. Essentially all of our study species, with the exception of gray-cheeked thrush and yellow warbler, nest on or near the ground, and these were two species that were particularly shrub-tolerant [25]. In addition to the availability of adequate nesting sites, birds require habitats that provide preferred food sources and types of foraging habitat. Research suggests that shrub dominance in tundra systems is linked with changes in insect abundance and species composition. In Alaskan tundra, shrub-dominated tundra had a greater biomass of canopy dwelling arthropods, whereas graminoid-dominated tundra sites had a greater biomass of ground-dwelling arthropods [24,50]. In addition to altering the composition of food sources, tall or dense vegetation may also hinder birds’ ability to forage effectively; therefore some birds may strongly select nesting or foraging habitat with shrubs but have limited tolerance for areas with particularly tall or dense shrubs [51]. The majority of our study species prefer to forage for insects on the ground and these birds may be at a disadvantage as shrub dominance increases beyond some threshold best signified by shrub height in this analysis.

Kessel [25] parsed upland bird habitat of the Seward Peninsula into categories defined by dominant vegetation types and prevailing shrub height and often described avian preferences for habitat based on shrub height. Our results largely corroborate the importance of shrub height to birds in this region: this metric was the most consistently supported predictor of habitat preference and had a large number of quadratic and exponential responses, indicating specific windows of tolerance for many species. Kessel [25] described American golden-plover, whimbrel, bristle-thighed curlew, western sandpiper, and Lapland longspur as species associated with the shortest shrubs or least shrubby habitats: dwarf shrub mat (dominated by woody plants <0.4 m) or dwarf shrub meadow (dominated by sedges and shrubs <0.4 m tall). We found all of these species to be particularly responsive to shrubs, with very narrow tolerances for at least one shrub metric. Kessel noted that willow ptarmigan and Savannah sparrow prefer low shrub thicket (dominated by shrubs 0.4–1.1 m tall) with intermingled or nearby herbaceous cover. Our results concur with Kessel’s assessment; both species were more abundant in habitats with higher herbaceous cover and reached near-zero predictions when average shrub height was >60 cm.

Our shrub metrics represented average shrub conditions within a 250-m radius around the survey point and thus did not reflect the heights of individual shrub patches available to birds within the sampled area. As a result, our average shrub heights are not directly comparable to the shrub categories described by Kessel [25]. For example, our shrub height metric was a combined average of the tallest shrub from four categories, combining low-growing understory shrubs across the area with shrubs that made up the prevailing canopy height; in contrast, Kessel’s shrub height estimates were based on visual estimates of average canopy heights of patches used by birds. Based on thresholds in predicted abundance, we estimate that Kessel’s cutoff between dwarf (40 cm), low (40–110 cm), medium (110–240 cm), and tall shrub thickets (240–490 cm) are equivalent to our average values of dwarf (10 cm), low (10–30 cm), medium (30–60 cm), and tall (>60 cm) shrubs. Studies investigating mechanistic relationships between shrub characteristics and bird abundance should be certain to account for the spatial scale at which such relationships are being measured.

Based on current avian habitat associations across subarctic tundra landscapes, our findings suggest that increases in shrub cover and density will negatively affect abundance of only a few bird species (arctic warbler, bristle-thighed curlew) and may potentially be beneficial for many (e.g. fox sparrow, bluethroat, Savannah sparrow, yellow warbler). As shrub height increases further, however, a considerable number of current tundra bird species will likely find preferred habitats (or “current habitats”) increasingly unsuitable. At this time we know very little about how tundra shrub characteristics and related geophysical and climactic factors might affect avian productivity and survival, the primary demographic processes that govern changes in breeding abundance. Increasing shrub cover or density may be beneficial to more generalist species, but may also signify changes in the timing, quality, quantity, or types of important foods, potentially attracting birds, but providing suboptimal conditions [52]. Increases in tall woody vegetation could draw in novel predators to which tundra birds may be ill-adapted, reducing survival or fecundity [53]. As avian assemblages shift from those preferring open tundra to more shrub-preferring communities, interspecific competitive forces may also affect productivity and survival of many species [54]. As in other regions, generalists are likely to benefit, at least in the near future [55].

## Supporting Information

S1 Fig Correlation assessment for four covariates used in analysis of bird abundance. (DOCX)

S1 Table Description of vegetation metrics recorded at point count stations. (DOCX)

S2 Table Summary of species recorded, ordered by total observations. (DOCX)

S3 Table ΔAIC values comparing five models for each habitat covariate relative to abundance. (DOCX)

S4 Table Coefficients and standard errors for habitat covariates in final models of abundance. (DOCX)

S5 Table Coefficients, standard errors, and p-values for covariates explaining detection probability (P_d_) in final models of bird abundance; estimated detection probability in average conditions. (DOCX)

## References

[1] Walther G-R, Post E, Convey P, Menzel A, Parmesan C, Beebee TJC, et al Ecological responses to recent climate change. Nature. 2002;416: 389–395. 10.1038/416389a 11919621

[2] Root TL, Price JT, Hall KR, Schneider SH, Rosenzweig C, Pounds JA. Fingerprints of global warming on wild animals and plants. Nature. 2003;421: 57–60. 10.1038/nature01333 12511952

[3] Tingley MW, Monahan WB, Beissinger SR, Moritz C. Birds track their Grinnellian niche through a century of climate change. Proc Natl Acad Sci U S A 2009;106: 19637–19643. 10.1073/pnas.0901562106 19805037 PMC2780944

[4] Serreze MC, Walsh JE, Chapin FS, Osterkamp TE, Dyurgerov MB, Romanovsky VE, et al Observational evidence of recent change in the northern high-latitude environment. Clim Change. 2000;46: 159–207.

[5] Solomon S, Qin D, Manning M, Chen Z, Marquis M, Averyt K, et al Climate change 2007: the physical science basis Contribution of Working Group I to the fourth assessment report of the Intergovernmental Panel on Climate Change. Cambridge, United Kingdom and New York: Cambridge University Press; 2007.

[6] Marcot BG, Jorgenson MT, Lawler JP, Handel CM, DeGange AR. Projected changes in wildlife habitats in arctic natural areas of northwest Alaska. Clim Change. 2015;130: 145–154.

[7] Rupp TS, Chapin FS, Starfield AM. Response of subarctic vegetation to transient climatic change on the Seward Peninsula in north-west Alaska. Glob Change Biol. 2000;6: 541–555.

[8] Chapin FS, Shaver GR, Giblin AE, Nadelhoffer KJ, Laundre JA. Responses of arctic tundra to experimental and observed changes in climate. Ecology. 1995;76: 694–711.

[9] Hinzman LD, Bettez ND, Bolton WR, Chapin FS, Dyurgerov MB, Fastie CL, et al Evidence and implications of recent climate change in northern Alaska and other arctic regions. Clim Change. 2005;72: 251–298.

[10] Hudson JMG, Henry GHR. Increased plant biomass in a High Arctic heath community from 1981 to 2008. Ecology. 2009;90: 2657–2663. 19886474 10.1890/09-0102.1

[11] Myers-Smith IH, Forbes BC, Wilmking M, Hallinger M, Lantz T, Blok D, et al Shrub expansion in tundra ecosystems: dynamics, impacts and research priorities. Environ Res Lett. 2011;6: 045509.

[12] Tape K, Sturm M, Racine C. The evidence for shrub expansion in northern Alaska and the Pan-Arctic. Glob Change Biol. 2006;12: 686–702.

[13] Dial RJ, Smeltz TS, Sullivan PF, Rinas CL, Timm K, Geck JE, et al Shrubline but not treeline advance matches climate velocity in montane ecosystems of south-central Alaska. Glob Change Biol. 2016;22: 1841–1856. 10.1111/gcb.13207 26719133

[14] Swanson JD, Schuman M, Scorup P. Range survey of the Seward Peninsula reindeer ranges, Alaska. U.S Dept. of Agriculture, Soil Conservation Service; 1985.

[15] Walker MD, Wahren CH, Hollister RD, Henry GHR, Ahlquist LE, Alatalo JM, et al Plant community responses to experimental warming across the tundra biome. Proc Natl Acad Sci U S A. 2006;103: 1342–1346. 10.1073/pnas.0503198103 16428292 PMC1360515

[16] Myers-Smith IH, Hik DS, Kennedy C, Cooley D, Johnstone JF, Kenney AJ, et al Expansion of canopy-forming willows over the twentieth century on Herschel Island, Yukon Territory, Canada. Ambio. 2011;40: 610–623. 10.1007/s13280-011-0168-y 21954724 PMC3357868

[17] Mizel JD, Schmidt JH, McIntyre CL, Roland CA. Rapidly shifting elevational distributions of passerine species parallel vegetation change in the subarctic. Ecosphere. 2016;7: e01264 10.1002/ecs2.1264

[18] MacArthur RH. Population ecology of some warblers of northeastern coniferous forests. Ecology. 1958;39: 599–619.

[19] Wiens JA, Rotenberry JT. Habitat associations and community structure of birds in shrubsteppe environments. Ecol Monogr. 1981;51: 21–41.

[20] Cody ML. Habitat selection in birds. Academic Press; 1985.

[21] Cunningham MA, Johnson DH. Proximate and landscape factors influence grassland bird distributions. Ecol Appl. 2006;16: 1062–1075. 16827003 10.1890/1051-0761(2006)016[1062:palfig]2.0.co;2

[22] Thompson SJ, Arnold TW, Amundson CL. A multiscale assessment of tree avoidance by prairie birds. Condor. 2014;116: 303–315.

[23] Grant TA, Madden E, Berkey GB. Tree and shrub invasion in northern mixed-grass prairie: implications for breeding grassland birds. Wildl Soc Bull. 2004;32: 807–818.

[24] Boelman NT, Gough L, Wingfield J, Goetz S, Asmus A, Chmura HE, et al Greater shrub dominance alters breeding habitat and food resources for migratory songbirds in Alaskan arctic tundra. Glob Change Biol. 2015;21: 1508–1520. 10.1111/gcb.12761 25294359

[25] Kessel B. Birds of the Seward Peninsula, Alaska. 1st ed Fairbanks: University of Alaska Press; 1989.

[26] Wahrhaftig C. Physiographic divisions of Alaska. US Government Printing Office. 1965. Available: http://pubs.usgs.gov/pp/0482/report.pdf

[27] Hutto RL, Pletschet SM, Hendricks P. A fixed-radius point count method for nonbreeding and breeding season use. Auk. 1986;103: 593–602.

[28] Buckland ST, Anderson DR, Burnham KP, Laake JL. Distance sampling. Encyclopedia of Biostatistics. 2005;2: 10.1002/0470011815.b2a16019

[29] Royle JA. N-mixture models for estimating population size from spatially replicated counts. Biometrics. 2004;60: 108–115. 10.1111/j.0006-341X.2004.00142.x 15032780

[30] Robel RJ, Briggs JN, Dayton AD, Hulbert LC. Relationships between visual obstruction measurements and weight of grassland vegetation. J Range Manag. 1970;23: 295–297.

[31] Zuur AF, Ieno EN, Smith GM. Analysing ecological data. New York: Springer; 2007.

[32] Fieberg J, Johnson DH. MMI: multimodel inference or models with management implications? J Wildl Manag. 2015;79: 708–718.

[33] Cottam G, Curtis JT. The use of distance measures in phytosociological sampling. Ecology. 1956;37: 451–460.

[34] Kéry M, Royle JA. Applied hierarchical modeling in ecology: analysis of distribution, abundance and species richness in R and BUGS Volume 1: Prelude and static models. 1st ed San Diego: Academic Press; 2015.

[35] Royle JA, Dawson DK, Bates S. Modeling abundance effects in distance sampling. Ecology. 2004;85: 1591–1597.

[36] Kéry M, Royle JA, Schmid H. Modeling avian abundance from replicated counts using binomial mixture models. Ecol Appl. 2005;15: 1450–1461.

[37] Chandler RB, Royle JA, King DI. Inference about density and temporary emigration in unmarked populations. Ecology. 2011;92: 1429–1435. 21870617 10.1890/10-2433.1

[38] Hocking DJ, Connette GM, Conner CA, Scheffers BR, Pittman SE, Peterman WE, et al Effects of experimental forest management on a terrestrial, woodland salamander in Missouri. For Ecol Manag. 2013;287: 32–39.

[39] Dail D, Madsen L. Models for estimating abundance from repeated counts of an open metapopulation. Biometrics. 2011;67: 577–587. 10.1111/j.1541-0420.2010.01465.x 20662829

[40] McNew LB, Handel CM. Evaluating species richness: biased ecological inference results from spatial heterogeneity in detection probabilities. Ecol Appl. 2015;25: 1669–1680. 26552273 10.1890/14-1248.1

[41] R Core Team. R: A language and environment for statistical computing. 2014. Available: https://www.r-project.org/

[42] Fiske I, Chandler R. unmarked: an R package for fitting hierarchical models of wildlife occurrence and abundance. J Stat Softw. 43(i10).

[43] Arnold TW. Uninformative parameters and model selection using Akaike’s information criterion. J Wildl Manag. 2010;74: 1175–1178.

[44] MacArthur RH, MacArthur JW. On bird species diversity. Ecology. 1961;42: 594–598.

[45] Zellweger F, Baltensweiler A, Ginzler C, Roth T, Braunisch V, Bugmann H, et al Environmental predictors of species richness in forest landscapes: abiotic factors versus vegetation structure. J Biogeogr. 2016;43: 1080–1090

[46] Kissling WD, Field R, Böhning-Gaese K. Spatial patterns of woody plant and bird diversity: functional relationships or environmental effects? Glob Ecol Biogeogr. 2008;17: 327–339.

[47] Stein A, Gerstner K, Kreft H. Environmental heterogeneity as a universal driver of species richness across taxa, biomes and spatial scales. Ecol Lett. 2014;17: 866–880. 10.1111/ele.12277 24751205

[48] Raynolds MK, Walker DA, Maier HA. Plant community-level mapping of arctic Alaska based on the Circumpolar Arctic Vegetation Map. Phytocoenologia. 2005;35: 821–848.

[49] Hussell DJT, Montgomerie R. Lapland Longspur (*Calcarius lapponicus*). Poole A, Gill F, editors. Birds N Am Online 2002;

[50] Rich ME, Gough L, Boelman NT. Arctic arthropod assemblages in habitats of differing shrub dominance. Ecography. 2013;36: 994–1003.

[51] Batova ON. Selection of foraging tactics in leaf warblers (*Phylloscopus*). Biol Bull. 2011;38: 259–265. 21789995

[52] Schlaepfer MA, Runge MC, Sherman PW. Ecological and evolutionary traps. Trends Ecol Evol. 2002;17: 474–480.

[53] Ballantyne K, Nol E. Nesting habitat selection and hatching success of whimbrels near Churchill, Manitoba, Canada. Waterbirds. 2011;34: 151–159.

[54] Julliard R, Clavel J, Devictor V, Jiguet F, Couvet D. Spatial segregation of specialists and generalists in bird communities. Ecol Lett. 2006;9: 1237–1244. 10.1111/j.1461-0248.2006.00977.x 17040326

[55] Davey CM, Chamberlain DE, Newson SE, Noble DG, Johnston A. Rise of the generalists: evidence for climate driven homogenization in avian communities. Glob Ecol Biogeogr. 2012;21: 568–578.

