# Supplementary material for: When Winners Become Losers: Predicted Nonlinear Responses of Arctic Birds to Increasing Woody Vegetation
Source: PLoS One. 2016 Nov 16;11(11):e0164755. doi: 10.1371/journal.pone.0164755 (PMC5112980; doi:10.1371/journal.pone.0164755)

**S1 Figure. Correlation assessment for four covariates used in analysis of bird abundance.** Numbers in upper half of panels show correlation coefficients, with size of text scaled to degree of correlation, and bottom panels show raw data with a loess smoother.


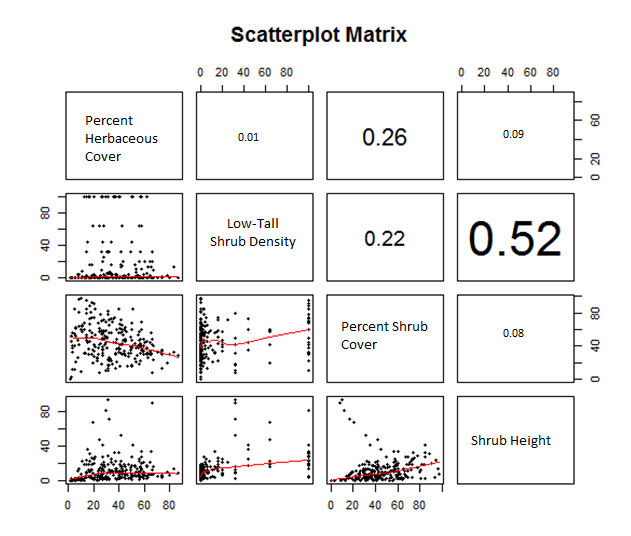

Supplement: S1 Fig — (DOCX) [file pone.0164755.s001.docx]
