# Supplementary material for: When Winners Become Losers: Predicted Nonlinear Responses of Arctic Birds to Increasing Woody Vegetation
Source: PLoS One. 2016 Nov 16;11(11):e0164755. doi: 10.1371/journal.pone.0164755 (PMC5112980; doi:10.1371/journal.pone.0164755)
Supplement: S1 Table — (DOCX) [file pone.0164755.s002.docx]

**S1 Table. Description of vegetation metrics recorded at point count stations.**

| Description of measurement | Scale^a^ | Min. | Mean | Max. | SD | Derivation or reason for excluding^b^ |
| --- | --- | --- | --- | --- | --- | --- |
| Distance (m) to dwarf shrub <0.5 m tall, average 4 quadrants | Truncated at 5 m | 0.0 | 0.5 | 5.0 | 1.2 | Small range, mostly 0s, highly skewed |
| Distance (m) to low shrubs (0.5 – 1.0 m tall), average 4 quadrants | Truncated at 110 m | 0.0 | 42.0 | 110.0 | 45.2 | Right skew, high collinearity |
| Distance (m) to tall shrubs (>1.0 m tall), average 4 quadrants | Truncated at 250 m | 0.0 | 142.9 | 250.0 | 107.1 | Right skew, high collinearity |
| **Shrub Density, low & tall shrubs, shrubs per 100 m^2^** | **Shrubs per 100 m^2^, from point count center** | **0.1** | **17.92** | **100.0** | **30.15** | **Generated by point-centered quadrat method = 100/((average distance in meters to shrub +1)^2). Added 1 to all average distances because distances <1 generate asymptotically large densities.** |
| Visual obstruction reading (dm), average | 10 sub-points | 0.0 | 1.6 | 5.0 | 1.2 | High collinearity |
| Percent lichen cover, average | 10 0.5x0.5 m^2^ sub-points | 0.0 | 15.8 | 77.3 | 17.2 | Collinear with visual obstruction (-0.63), Height herbaceous (-0.63) |
| Percent bare ground, average | 10 0.5x0.5 m^2^ sub-points | 0.0 | 7.0 | 97.5 | 15.1 | Right skew, mostly 0s |
| Percent willow cover, average | 10 0.5x0.5 m^2^ sub-points | 0.0 | 10.9 | 78.3 | 12.9 | Combined; decent distribution, right skew |
| Percent alder cover, average | 10 0.5x0.5 m^2^ sub-points | 0.0 | 2.0 | 82.3 | 10.8 | Combined; only 15 non-zero values |
| Percent dwarf birch cover, average | 10 0.5x0.5 m^2^ sub-points | 0.0 | 6.8 | 60.3 | 8.0 | Combined; decent distribution, right skew |
| Percent ericaceous shrub cover, average | 10 0.5x0.5 m^2^ sub-points | 0.0 | 26.9 | 85.3 | 16.2 | Combined; good distribution, few missing values/0s |
| **Shrub cover, average, combined** | **10 0.5x0.5 m^2^ sub-points** | **0**.0 | **44.4** | **97.5** | **19.4** | **Sum of average percent willow, alder, dwarf birch, and ericaceous shrub** |
| **Combined average shrub height (cm)** | **10 0.5x0.5 m^2^ sub-points** | **0.0** | **12.0** | **94.1** | **13.5** | **Overall average of all available (i.e., excluding NA) shrub heights (range 0-39 measurements per point due to NA values when no shrub of that group was within the 0.5 m subplot); truncated at 100 cm, reducing 8 high values.** |
| Height (cm) of tallest dwarf birch, average | 10 0.5x0.5 m^2^ sub-points | 4.0 | 22.9 | 72.2 | 13.6 | Combined; many 0s and right skew |
| Height (cm) of tallest willow, average | 10 0.5x0.5 m^2^ sub-points | 1.0 | 29.8 | 181.0 | 33.4 | Combined; good distribution |
| Height (cm) of tallest alder, average | 10 0.5x0.5 m^2^ sub-points | 1.0 | 203.1 | 398.0 | 140.3 | Combined; only 15 non-NA values |
| Height (cm) of tallest ericaceous shrub, average | 10 0.5x0.5 m^2^ sub-points | 2.4 | 14.1 | 40.5 | 8.1 | Combined; good distribution |
| **Percent herbaceous cover, average** | **10 0.5x0.5 m**^2^ **sub-points** | **1.3** | **36.5** | **86.5** | **19.3** | **Included** |
| Height (cm) of tallest herbaceous plant, average | 10 0.5x0.5 m^2^ sub-points | 5.0 | 26.5 | 59.4 | 10.7 | High collinearity |

Bold font indicates variables that were included in models. ‘NA’ indicates values not recorded because vegetation type was not present at survey point.

^a^ ‘Scale’ indicates the scope or size of the measurement: (1) distance from survey point at which measurements to nearest shrub of given height class were truncated; (2) measurements taken at 10 sub-points, 5 on each of 2 or 3 randomly-oriented 20-m transects, one emanating from survey point and the other(s) within 250 m of survey point; (3) percent cover recorded within ten 0.5 x 0.5 m^2^ subsample quadrats at same points as previous.

^b^ The final column briefly (1) details the reason a variable was not included in the analysis or (2) describes how an included variable was derived from other metrics. Reasons for exclusion were (1) variable was ultimately combined with other measurements and included; (2) variable was poorly distributed; often highly skewed, or consisted of predominantly zeros or missing values; or (3) variable was strongly collinear with other measurements that were included.
