# Supplementary material for: When Winners Become Losers: Predicted Nonlinear Responses of Arctic Birds to Increasing Woody Vegetation
Source: PLoS One. 2016 Nov 16;11(11):e0164755. doi: 10.1371/journal.pone.0164755 (PMC5112980; doi:10.1371/journal.pone.0164755)
Supplement: S2 Table — (DOCX) [file pone.0164755.s003.docx]

**S2 Table. Summary of species recorded, ordered by total observations.**

| Common Name | Scientific Name | 2012 | 2013 | 2014 | Total |
| --- | --- | --- | --- | --- | --- |
| Lapland longspur | *Calcarius lapponicus* | 347 | 733 | 564 | 1644 |
| Hoary redpoll | *Acanthis hornemanni* | 219 | 451 | 708 | 1378 |
| Savannah sparrow | *Passerculus sandwichensis* | 87 | 540 | 537 | 1164 |
| Fox sparrow | *Passerella iliaca* | 109 | 372 | 422 | 903 |
| Golden-crowned sparrow | *Zonotrichia atricapilla* | 150 | 231 | 164 | 545 |
| Gray-cheeked thrush | *Catharus minimus* | 40 | 179 | 228 | 447 |
| American tree sparrow | *Spizelloides arborea* | 12 | 192 | 140 | 344 |
| Wilson's snipe | *Gallinago delicata* | 66 | 133 | 104 | 303 |
| Bluethroat | *Luscinia svecica* | 26 | 119 | 141 | 286 |
| American robin | *Turdus migratorius* | 22 | 106 | 127 | 255 |
| Long-tailed jaeger | *Stercorarius longicaudus* | 92 | 104 | 57 | 253 |
| Arctic warbler | *Phylloscopus borealis* | 2 | 128 | 122 | 252 |
| Yellow warbler | *Setophaga petechia* | 28 | 83 | 126 | 237 |
| American golden-plover | *Pluvialis dominica* | 27 | 81 | 85 | 193 |
| White-crowned sparrow | *Zonotrichia leucophrys* | 20 | 66 | 107 | 193 |
| Willow ptarmigan | *Lagopus lagopus* | 37 | 115 | 30 | 182 |
| Western sandpiper | *Calidris mauri* | 29 | 99 | 43 | 171 |
| Orange-crowned warbler | *Oreothlypis celata* | 4 | 57 | 92 | 153 |
| Wilson's warbler | *Cardellina pusilla* | 3 | 36 | 111 | 150 |
| Common redpoll | *Acanthis flammea* | 19 | 29 | 99 | 147 |
| Whimbrel | *Numenius phaeopus* | 9 | 44 | 66 | 119 |
| Pacific golden-plover | *Pluvialis fulva* | 21 | 62 | 33 | 116 |
| American pipit | *Anthus rubescens* | 40 | 45 | 26 | 111 |
| Northern waterthrush | *Parkesia noveboracensis* | 7 | 8 | 85 | 100 |
| Bristle-thighed curlew | *Numenius tahitiensis* | 4 | 35 | 54 | 93 |
| Bar-tailed godwit | *Limosa lapponica* | 3 | 28 | 25 | 56 |
| Northern wheatear | *Oenanthe oenanthe* | 20 | 22 | 11 | 53 |
| Varied thrush | *Ixoreus naevius* | 0 | 0 | 51 | 51 |
| Rock ptarmigan | *Lagopus muta* | 6 | 37 | 7 | 50 |
| Common raven | *Corvus corax* | 20 | 11 | 11 | 42 |
| Blackpoll warbler | *Setophaga striata* | 2 | 2 | 31 | 35 |
| Horned lark | *Eremophila alpestris* | 6 | 11 | 13 | 30 |
| Unknown redpoll | *Acanthis* spp. | 0 | 12 | 14 | 26 |
| Canada goose | *Branta canadensis* | 2 | 14 | 5 | 21 |
| Dunlin | *Calidris alpina* | 1 | 19 | 0 | 20 |
| Pectoral sandpiper | *Calidris melanotos* | 8 | 0 | 6 | 14 |
| Snow bunting | *Plectrophenax nivalis* | 6 | 7 | 1 | 14 |
| Surfbird | *Calidris virgata* | 0 | 12 | 2 | 14 |
| Sandhill crane | *Grus canadensis* | 0 | 13 | 0 | 13 |
| Glaucous gull | *Larus hyperboreus* | 7 | 3 | 1 | 11 |
| Northern pintail | *Anas acuta* | 5 | 2 | 4 | 11 |
| Rough-legged hawk | *Buteo lagopus* | 1 | 4 | 6 | 11 |
| Parasitic jaeger | *Stercorarius parasiticus* | 2 | 5 | 3 | 10 |
| Eastern yellow wagtail | *Motacilla tschutschensis* | 4 | 1 | 2 | 7 |
| Black-bellied plover | *Pluvialis squatarola* | 6 | 0 | 0 | 6 |
| Northern harrier | *Circus cyaneus* | 2 | 3 | 1 | 6 |
| Semipalmated sandpiper | *Calidris pusilla* | 4 | 2 | 0 | 6 |
| Short-eared owl | *Asio flammeus* | 3 | 0 | 2 | 5 |
| Gray jay | *Perisoreus canadensis* | 0 | 0 | 4 | 4 |
| Ruby-crowned kinglet | *Regulus calendula* | 0 | 0 | 4 | 4 |
| Tundra swan | *Cygnus columbianus* | 2 | 2 | 0 | 4 |
| Red-breasted merganser | *Mergus serrator* | 0 | 3 | 0 | 3 |
| Spotted sandpiper | *Actitis macularius* | 0 | 0 | 3 | 3 |
| Yellow-rumped warbler | *Setophaga coronata* | 0 | 1 | 2 | 3 |
| Bank swallow | *Riparia riparia* | 0 | 0 | 2 | 2 |
| Greater white-fronted goose | *Anser albifrons* | 0 | 0 | 2 | 2 |
| Long-tailed duck | *Clangula hyemalis* | 2 | 0 | 0 | 2 |
| Merlin | *Falco columbarius* | 0 | 1 | 1 | 2 |
| Red knot | *Calidris canutus* | 0 | 0 | 2 | 2 |
| Rock pigeon | *Columba livia* | 0 | 0 | 2 | 2 |
| Say's phoebe | *Sayornis saya* | 0 | 0 | 2 | 2 |
| Semipalmated plover | *Charadrius semipalmatus* | 1 | 0 | 1 | 2 |
| Buff-breasted sandpiper | *Calidris subruficollis* | 0 | 1 | 0 | 1 |
| Boreal chickadee | *Poecile hudsonicus* | 0 | 0 | 1 | 1 |
| Golden eagle | *Aquila chrysaetos* | 1 | 0 | 0 | 1 |
| Glaucous-winged gull | *Larus glaucescens* | 0 | 1 | 0 | 1 |
| Long-billed dowitcher | *Limnodromus scolopaceus* | 0 | 0 | 1 | 1 |
| Mallard | *Anas platyrhynchos* | 0 | 1 | 0 | 1 |
