# Supplementary material for: When Winners Become Losers: Predicted Nonlinear Responses of Arctic Birds to Increasing Woody Vegetation
Source: PLoS One. 2016 Nov 16;11(11):e0164755. doi: 10.1371/journal.pone.0164755 (PMC5112980; doi:10.1371/journal.pone.0164755)
Supplement: S3 Table — (DOCX) [file pone.0164755.s004.docx]

**S3 Table. ΔAIC values comparing five models for each habitat covariate relative to abundance.**

| Species | Average Shrub Height | | | | | Shrub Density (per 100 m^2^) | | | | | Shrub Cover (%) | | | | | Herbaceous Cover (%) | | | | |
| --- | --- | --- | --- | --- | --- | --- | --- | --- | --- | --- | --- | --- | --- | --- | --- | --- | --- | --- | --- | --- |
|  | *Null* | *Lin* | *Exp* | *Nexp* | *Quad* | *Null* | *Lin* | *Exp* | *Nexp* | *Quad* | *Null* | *Lin* | *Exp* | *Nexp* | *Quad* | *Null* | *Lin* | *Exp* | *Nexp* | *Quad* |
| American golden-plover | 52.6 | 6.0 | 36.6 | **0.0** | 3.6 | **0.0** | 1.2 | 1.4 | 1.1 | 3.4 | 4.8 | 3.2 | 6.4 | **0.0** | 3.1 | **0.0** | 2.2 | 2.3 | 2.0 | 4.3 |
| Arctic warbler | 7.1 | 8.7 | 5.2 | 5.7 | **0.0** | 2.8 | **1.6** | 2.9 | **0.0** | 2.7 | **0.4** | 2.2 | 2.7 | **0.0** | 2.6 | 9.7 | 6.6 | **0.7** | 11.0 | **0.0** |
| American tree sparrow | 29.1 | 26.5 | 16.6 | 30.0 | **0.0** | 7.9 | 6.0 | 8.9 | **0.8** | **0.0** | **1.5** | 2.4 | **0.0** | 3.7 | 1.0 | **0.0** | 2.1 | 2.1 | 1.2 | 1.5 |
| Bluethroat | **0.9** | **0.0** | 2.4 | 3.1 | **0.5** | 5.0 | 4.0 | 5.5 | **0.0** | 2.3 | 3.3 | **0.0** | 0.6 | 2.8 | 1.9 | **0.0** | 2.2 | 1.5 | 1.6 | 1.9 |
| Bristle-thighed curlew | 4.0 | **2.4** | **0.0** | 5.0 | 2.8 | **0.0** | 1.2 | 1.1 | 1.7 | 3.3 | 8.4 | **0.0** | 3.9 | 3.3 | 2.2 | **1.3** | **0.0** | **0.4** | **1.3** | 2.2 |
| Fox sparrow | 4.7 | **0.0** | 3.7 | 3.9 | 2.1 | 7.4 | 8.9 | 9.6 | 4.2 | **0.0** | 17.8 | **0.0** | 5.4 | 9.6 | 1.9 | 3.2 | 2.9 | **0.0** | 4.9 | 1.7 |
| Golden-crowned sparrow | 10.0 | **0.0** | 9.9 | **0.7** | **1.0** | **1.8** | **1.5** | 2.1 | **0.0** | 2.9 | 29.5 | **0.0** | 15.0 | 5.1 | 1.7 | **0.0** | 2.2 | 1.4 | 1.7 | 1.7 |
| Gray-cheeked thrush | 4.5 | **0.0** | 3.6 | 3.0 | 2.2 | **0.0** | 2.2 | 2.1 | 1.3 | 1.3 | 4.2 | **0.0** | 2.5 | 2.1 | 2.2 | 3.4 | **0.0** | **1.1** | 2.1 | 2.1 |
| Lapland longspur | 50.8 | 4.2 | **2.0** | 24.2 | **0.0** | 15.8 | 9.5 | 14.9 | **0.0** | **0.0** | **0.0** | 2.2 | 1.9 | 1.0 | 2.0 | 19.9 | **0.0** | 6.7 | 7.7 | 2.2 |
| Northern waterthrush | 17.4 | 7.6 | 17.7 | **0.0** | 4.5 | **0.0** | 1.9 | 2.1 | 1.9 | 4.0 | **0.0** | 0.9 | 0.5 | 1.4 | 3.1 | 8.0 | **0.0** | **1.0** | 4.9 | 1.4 |
| Savannah sparrow | 31.9 | 22.2 | 5.6 | 34.1 | **0.0** | **0.0** | 2.1 | 2.2 | 1.3 | 2.6 | 5.4 | **0.0** | 1.9 | 2.8 | 2.1 | 6.7 | **1.2** | 6.5 | **0.0** | 2.0 |
| White-crowned sparrow | 14.1 | 13.8 | 15.4 | **0.0** | 0.7 | **0.0** | 2.1 | 2.0 | 2.2 | 3.9 | 2.9 | **0.0** | 0.5 | 3.2 | 2.0 | **0.0** | 2.2 | 2.1 | 2.2 | 3.9 |
| Western sandpiper | 17.4 | **0.0** | 6.0 | **0.1** | 1.6 | **0.0** | 2.0 | 2.2 | 1.9 | 4.0 | **0.4** | 2.6 | **0.6** | 2.2 | **0.0** | 8.6 | 2.9 | 7.8 | **0.0** | 3.5 |
| Whimbrel | **1.9** | 3.8 | **1.6** | 3.7 | **0.0** | **0.0** | 2.2 | 2.2 | 2.2 | 4.2 | 2.9 | 4.7 | 2.8 | 4.6 | **0.0** | **0.0** | 2.1 | 0.5 | 2.3 | 2.2 |
| Willow ptarmigan | 9.1 | **0.9** | **0.0** | 6.1 | 2.1 | **0.0** | 1.0 | 1.1 | 0.4 | 3.1 | **0.0** | 1.8 | 1.9 | 2.0 | 4.1 | 9.7 | **0.5** | 8.3 | **0.0** | 0.9 |
| Wilson’s warbler | 6.8 | **0.4** | 8.2 | **0.0** | 0.1 | **0.8** | **1.1** | **0.0** | 2.5 | 1.5 | **1.5** | **0.0** | **0.3** | 2.1 | 2.0 | **0.5** | **1.4** | **0.0** | 2.3 | 2.2 |
| Yellow warbler | **1.8** | **0.0** | **1.6** | **1.4** | 2.2 | **0.3** | **1.3** | **1.9** | **0.0** | 2.0 | **0.2** | **0.0** | **0.3** | **0.7** | 2.3 | 10.4 | 2.9 | **0.0** | 9.8 | 0.4 |

For each bird species, we compared five models to test the relationship between abundance and each habitat covariate: null (covariate excluded), linear (lin), exponential (exp), negative exponential (negexp), and quadratic (quad). When testing for best fit of a particular covariate, we included all other habitat covariates as quadratic terms. The fit with the lowest AIC for each variable was then used for the top model. Top models (ΔAIC = 0.0) and competitive models (ΔAIC ≤ 2.0) are highlighted in bold except in the case of nested models with uninformative parameters.
