# Supplementary material for: When Winners Become Losers: Predicted Nonlinear Responses of Arctic Birds to Increasing Woody Vegetation
Source: PLoS One. 2016 Nov 16;11(11):e0164755. doi: 10.1371/journal.pone.0164755 (PMC5112980; doi:10.1371/journal.pone.0164755)
Supplement: S4 Table — (DOCX) [file pone.0164755.s005.docx]

**S4 Table. Coefficients and standard errors for habitat covariates in final models of abundance.**

| Species | Intercept | ShrubHeight | ShrubHeight ^2^ | ShrubDense | ShrubDense ^2^ | ShrubCover | ShrubCover ^2^ | HerbCover | HerbCover ^2^ |
| --- | --- | --- | --- | --- | --- | --- | --- | --- | --- |
| American golden-plover | -0.515, 1.999 | 2.328, 0.267 (NE) | NA | NA | NA | -0.235, 0.087 (NE) | NA | NA | NA |
| Arctic warbler | 0.484, 0.329 | 0.809, 0.291 | -0.251, 0.084 | -0.545 0.217 (NE) | NA | 0.191, 0.113 (NE) | NA | -0.334, 0.150 | -0.433, 0.169 |
| American tree sparrow | 0.184, 0.150 | 1.116, 0.285 | -0.696, 0.169 | 0.835 0.241 | -0.264 0.092 | 0.053, 0.030 (E) | NA | NA | NA |
| Bluethroat | 0.784, 0.270 | -0.177, 0.118 | NA | -0.651 0.174 (NE) | NA | 0.265, 0.084 | NA | NA | NA |
| Bristle-thighed curlew | 2.454, 1.113 | *-0.064, 0.069* (E) | NA | NA | NA | -0.681, 0.182 | NA | -0.345, 0.166 | NA |
| Fox sparrow | 0.779, 0.112 | 0.188, 0.054 | NA | 0.612 0.166 | -0.225 0.065 | 0.271, 0.057 | NA | -0.105, 0.045 (E) | NA |
| Golden-crowned sparrow | 0.639, 0.291 | -0.611, 0.183 | NA | -0.384 0.207 (NE) | NA | 0.554, 0.089 | NA | NA | NA |
| Gray-cheeked thrush | 0.112, 0.130 | 0.272, 0.053 | NA | NA | NA | 0.222, 0.071 | NA | -0.125, 0.063 (E) | NA |
| Lapland longspur | 1.005, 0.160 | -0.956, 0.142 | -0.518, 0.215 | -0.932 0.240 | 0.326 0.102 | NA | NA | 0.242 0.041 | NA |
| Northern waterthrush | 0.790, 0.407 | -2.139, 0.367 (E) | NA | NA | NA | NA | NA | NA | NA |
| Savannah sparrow | 1.249, 0.095 | 0.297, 0.125 | -0.429, 0.107 | NA | NA | 0.168, 0.064 | NA | -0.137, 0.047 (NE) | NA |
| White-crowned sparrow | 0.641, 0.261 | -1.110, 0.206 (NE) | NA | NA | NA | 0.419, 0.097 | NA | NA | NA |
| Western sandpiper | 3.304, 0.961 | -2.181, 0.450 | NA | NA | NA | -0.169, 0.211 | -0.366, 0.182 | -0.360, 0.115 (NE) | NA |
| Whimbrel | 4.153, 0.810 | 0.106, 0.360 | -1.289, 0.610 | NA | NA | -0.048, 0.233 | -0.600, 0.259 | NA | NA |
| Willow ptarmigan | 1.621, 0.556 | -0.275, 0.128 (E) | NA | NA | NA | NA | NA | -0.306, 0.084 (NE) | NA |
| Wilson’s warbler | 1.097, 0.305 | -1.136, 0.216 (NE) | NA | *-0.030, 0.022 (E)* | *NA* | *0.084, 0.106* | *NA* | -0.202, 0.098 (E) | NA |
| Yellow warbler | 0.512, 0.365 | 0.310, 0.122 | NA | -0.449, 0.247 (NE) | NA | 0.184, 0.124 | NA | -0.418, 0.131 (E) | NA |

See S1 Table for details on covariates. ‘NA’ indicates covariate was not included in final model. Abbreviations in parentheses indicate the type of relationship, if not linear, that best fit the predicted association between habitat covariate and abundance: (NE) = negative exponential transformation; (E) = exponential transformation. Coefficients in gray and italics were not significantly supported under the criteria of AIC-based model selection (i.e. p-values >0.15).
