# Supplementary material for: When Winners Become Losers: Predicted Nonlinear Responses of Arctic Birds to Increasing Woody Vegetation
Source: PLoS One. 2016 Nov 16;11(11):e0164755. doi: 10.1371/journal.pone.0164755 (PMC5112980; doi:10.1371/journal.pone.0164755)
Supplement: S5 Table — (DOCX) [file pone.0164755.s006.docx]

**S5 Table. Coefficients, standard errors, and p-values for covariates explaining detection probability (P_d_) in final models of bird sbundance; rstimated detection probability in average conditions.**

|  | Intercept | Date^a^ | Wind^b^ | Hour^c^ | Hour^2^ | P_d_^d^ | SE of P_d_^e^ |
| --- | --- | --- | --- | --- | --- | --- | --- |
| American golden-plover | -2.189 (2.66), 0.41 | -0.010 (0.02), 0.56 | -0.002 (0.02), 0.93 | -0.133 (0.28), 0.64 | -0.002 (0.01), 0.87 | 0.182 | 0.039 |
| Arctic warbler | -4.623 (3.38), 0.17 | 0.203 (0.04), 0 | -0.097 (0.03), 0 | 0.093 (0.62), 0.88 | -0.009 (0.03), 0.75 | 0.156 | 0.035 |
| American tree sparrow | -5.083 (1.88), 0.01 | 0.101 (0.02), 0 | -0.071 (0.03), 0.01 | 0.7 (0.37), 0.06 | -0.037 (0.02), 0.04 | 0.437 | 0.050 |
| Bluethroat | -3.41 (1.69), 0.04 | -0.042 (0.02), 0.03 | -0.045 (0.02), 0.06 | 0.887 (0.32), 0.01 | -0.047 (0.01), 0 | 0.427 | 0.057 |
| Bristle-thighed curlew | -2.454 (2.35), 0.31 | 0.014 (0.02), 0.56 | -0.008 (0.03), 0.77 | -0.22 (0.36), 0.54 | 0.000 (0.02), 0.97 | 0.121 | 0.036 |
| Fox sparrow | -2.733 (1.46), 0.06 | -0.029 (0.02), 0.1 | -0.086 (0.02), 0 | 1.045 (0.27), 0 | -0.058 (0.01), 0 | 0.654 | 0.032 |
| Golden-crowned sparrow | -2.961 (1.65), 0.07 | 0.014 (0.02), 0.54 | -0.06 (0.02), 0 | 0.679 (0.29), 0.02 | -0.036 (0.01), 0.01 | 0.500 | 0.058 |
| Gray-cheeked thrush | -6.153 (1.94), 0.00 | 0.081 (0.02), 0 | -0.108 (0.03), 0 | 1.145 (0.36), 0 | -0.057 (0.02), 0 | 0.561 | 0.042 |
| Lapland longspur | 1.178 (0.78), 0.13 | -0.012 (0.01), 0.2 | -0.007 (0.01), 0.46 | -0.189 (0.12), 0.12 | 0.005 (0.01), 0.31 | 0.532 | 0.030 |
| Northern waterthrush | 0.125 (3.33), 0.97 | -0.126 (0.05), 0.01 | -0.1 (0.05), 0.05 | 0.571 (0.59), 0.33 | -0.033 (0.03), 0.22 | 0.332 | 0.120 |
| Savannah sparrow | -1.81 (1.09), 0.1 | 0.043 (0.01), 0 | -0.049 (0.02), 0 | 0.364 (0.2), 0.07 | -0.021 (0.01), 0.02 | 0.557 | 0.034 |
| White-crowned sparrow | 3.5 (1.94), 0.07 | -0.056 (0.02), 0.01 | -0.048 (0.03), 0.13 | -0.302 (0.35), 0.39 | 0.003 (0.02), 0.86 | 0.318 | 0.060 |
| Western sandpiper | -4.956 (2.02), 0.01 | 0.008 (0.02), 0.70 | -0.010 (0.02), 0.69 | 0.159 (0.32), 0.61 | -0.012 (0.01), 0.37 | 0.184 | 0.036 |
| Whimbrel | 1.728 (2.23), 0.44 | -0.103 (0.03), 0.00 | 0.032 (0.03), 0.28 | -0.511 (0.39), 0.19 | 0.001 (0.02), 0.96 | 0.086 | 0.027 |
| Willow ptarmigan | 3.509 (2.28), 0.12 | -0.115 (0.02), 0.00 | 0 (0.02), 0.98 | -0.322 (0.33), 0.33 | 0.001 (0.01), 0.97 | 0.143 | 0.048 |
| Wilson’s warbler | -3.749 (2.83), 0.19 | -0.007 (0.03), 0.8 | -0.081 (0.03), 0.01 | 0.753 (0.51), 0.14 | -0.036 (0.02), 0.14 | 0.341 | 0.066 |
| Yellow warbler | -5.31 (2.37), 0.02 | 0.101 (0.03), 0 | -0.02 (0.03), 0.51 | 0.639 (0.42), 0.12 | -0.029 (0.02), 0.14 | 0.475 | 0.067 |

^a^ Date is the chronological day of survey with 1 = May 22^nd^.

^b^ Wind measured in km h^-1^.

^c^ Hour of the day (0-23) during which the survey took place in Alaska Daylight Time.

^d^ P_d_ is the estimated probability of detecting a bird during a 10-min survey given average survey conditions: 8 km h^-1^ wind, survey conducted on June 11 at 1000.

^e^ Standard error of estimated P_d_ during average survey conditions.
